# Wildlife, Exotic Pets, and Emerging Zoonoses^1^

**DOI:** 10.3201/eid1301.060480

**Published:** 2007-01

**Authors:** Bruno B. Chomel, Albino Belotto, François-Xavier Meslin

**Affiliations:** * School of Veterinary Medicine, University of California, Davis, Davis, California, USA; † Pan American Health Organization, Washington, DC, USA; ‡ World Health Organization, Geneva, Switzerland

**Keywords:** Emerging zoonoses, wildlife, ecotourism, exotic pets, synopsis

## Abstract

Most emerging infectious diseases are zoonotic; wildlife constitutes a large and often unknown reservoir. Wildlife can also be a source for reemergence of previously controlled zoonoses. Although the discovery of such zoonoses is often related to better diagnostic tools, the leading causes of their emergence are human behavior and modifications to natural habitats (expansion of human populations and their encroachment on wildlife habitat), changes in agricultural practices, and globalization of trade. However, other factors include wildlife trade and translocation, live animal and bushmeat markets, consumption of exotic foods, development of ecotourism, access to petting zoos, and ownership of exotic pets. To reduce risk for emerging zoonoses, the public should be educated about the risks associated with wildlife, bushmeat, and exotic pet trades; and proper surveillance systems should be implemented.

## Abstract 2

Wildlife and exotic pets represent large reservoirs for emerging zoonoses.

Emerging and reemerging infectious diseases have received increasing attention since the end of the 20th century. An estimated 75% of emerging infectious diseases are zoonotic, mainly of viral origin, and likely to be vectorborne (*1*). The emergence and rapid spread of West Nile virus in North America and the monkeypox outbreak in pet prairie dogs have been major awakening public health events that underscored the need for closer collaboration between the veterinary profession, wildlife specialists, and public health personnel (*2*,*3*). These events emphasized the role that veterinarians and other wildlife specialists can play in surveillance, control, and prevention of emerging zoonoses, as their training in disease recognition and population medicine makes them well suited for early detection networks (*4*).

Infectious pathogens of wildlife affect not only human health and agricultural production but also wildlife-based economies and wildlife conservation. Zoonotic pathogens that infect domestic animals and wildlife hosts are more likely to emerge (*5*). Furthermore, our quest for close contact with wild animals and for exotic pets puts us at risk for exposure to zoonoses.

## Economic Effects of Wildlife

Wildlife is a major source of income, either directly for consumptive or productive use value or indirectly for touristic and scientific values (*6*). For instance, wildlife tourism is among the top exporting activities of Tanzania and Kenya and generates an annual income of approximately half a billion US dollars (*6*). Even in industrialized countries, wildlife-related activities can generate major income. In the United States, the total expenditure for wildlife-related activities was $101 billion in 1996, ≈1.4% of the national economy (*6*). Hunting activities for the 10 million hunters in Europe generate a financial flux of almost 10 billion euros and ≈100,000 jobs. Europe is also the world’s largest importer of venison (>50,000 tons/year). Similarly, in the United States, hunting activities generate >700,000 jobs (*6*). In Africa, the bushmeat trade is generating hundreds of millions of dollars (*7*). In the Congo Basin, trade and regional consumption of wild animal meat could reach 4.5 million tons annually; the demand for bushmeat in western and Central Africa could reach up to 4× the demand for bushmeat in the Amazon Basin (*8*). Worldwide, deer farming has been developing dramatically. In New Zealand, ≈2 million farmed deer, half of the world’s farmed deer population, generate an annual income of NZ $200 million (*6*).

## Human Population Expansion and Encroachment on Wildlife Habitat

The exponential growth of the human population, from ≈1 billion in 1900 to 6.5 billion in 2006, has led to major ecologic changes and drastic wildlife habitat reduction. Many examples of the emergence or reemergence of zoonoses related to human encroachment on wildlife habitats exist.

Deforestation, development of human habitat, and mining activities have been suggested as risk factors associated with the reemergence of vampire bat rabies in humans in the Amazon Basin. In 2004, 46 persons died of rabies transmitted by vampire bats, mainly in Brazil (22 cases) and Colombia (14 cases); only 20 human cases of rabies were transmitted by dogs in all Latin America (*9*). A similar trend was again observed for 2005.

When first described in 1957, Kyasanur Forest disease was restricted to a much smaller area (300 square miles) in India than the actual 2,000 square miles of endemic zone (*10*). This tickborne disease occurs in evergreen rain forests interspersed with deciduous patches and clearings for rice cultivation and human habitations. Forest workers are particularly at risk; their mortality rates may reach 10%. In 1983, a major epidemic occurred during which several monkeys died, 1,555 humans were infected, and 150 humans died. The outbreak occurred in previously undisturbed forest where some 400 ha were clearcut to establish a cashew tree plantation. Most of the human patients were immigrant laborers employed to clear the forest (*10*). As many as 1,000 human cases occur each year, and this number has increased in the past 5 years. Most cases occur during the dry season (January–May), when nymphal activity is maximal. Such a zoonosis is a good example of deforestation and agricultural development leading to human habitat expansion into natural foci of a viral infection. Because cleared areas were widely used for grazing of cattle, a major host for adult ticks, these areas favored the proliferation of the tick *Haemaphysalis spinigera.*

Conversely, the reduction of traditional agricultural land and its replacement with forested areas, home to the main reservoirs and hosts of *Borrelia burgdorferi*, in association with the settlement of persons in periurban areas led to a considerable increase in human cases of Lyme disease in the United States (*11*). An estimated 32.4 million wild ruminants, major amplifiers for adult *Ixodes scapularis* ticks, live in North America (*12*). An estimated 23–40 million white-tailed deer inhabited North America before the arrival of Europeans; the deer population was greatly reduced by habitat loss and unrestricted hunting. However, by the mid-20th century, the population was restored throughout North America, and an estimated 14–20 million white-tailed deer are believed to inhabit the United States alone. In many areas of the eastern United States, populations have soared to previously unattained levels (http://www.aphis.usda.gov/ws/nwrc/is/living/deer.pdf).

Human activities may also be a source of wildlife infection, which could create new reservoirs of human pathogens. The recent outbreak of tuberculosis caused by *Mycobacterium tuberculosis* in suricats and mongooses was one of the first documented spillovers of a human disease within a wildlife population (*13*). Banded mongooses were observed feeding regularly at garbage pits and were therefore exposed to human excretions and any infectious material from tuberculosis-infected humans.

## Changes in Agricultural Practices and Emergence of Wildlife Zoonoses

The emergence of Argentine hemorrhagic fever in east-central Argentina during the 1950s, and its expansion to north-central Argentina, has been directly linked to development of agricultural activities (mainly corn growing) that sustain the virus’s main reservoir, the corn mouse (*Calomys musculinus*). Caused by the Junin virus, Argentine hemorrhagic fever affects primarily adult male agricultural workers, mainly during the harvest season (*14*).

In the late 1970s and early 1980s, a rabies epidemic occurred in free-ranging greater kudus (*Tragelaphus strepsiceros*) in Namibia (*15*). The kudu population had increased considerably in response to favorable conditions and human-made environmental changes. Suitable conditions for transmission in the kudu population after initial infection by rabid carnivores are provided by the social behavior of kudus, such as browsing on thorny acacia trees and resultant lesions in the kudus’ oral cavity, and excretion of relatively high titers of virus in the saliva of infected animals (*15*).

The outbreak of Nipah virus infection in Malaysia during 1998–1999, which caused 265 human cases of viral encephalitis and a 38% mortality rate, was also the result of several major ecologic and environmental changes associated with deforestation and expansion of nonindustrial pig farming in association with production of fruit-bearing trees (*16*). Such combination led to infection of pigs, which developed respiratory and neurologic symptoms after indirect exposure to infected fruit bats that shed the virus. The sick pigs were a subsequent source of human infection (*16*).

Farming of wild animal species led to reemergence of zoonoses such as bovine tuberculosis in captive deer populations. Deer at low population densities on natural range are less likely to be affected to any major extent by disease. However, disease becomes a factor in intensive management of deer (*17*). Reemergence of zoonotic diseases that had been controlled from their domestic animal reservoirs is also of major concern. Wildlife may become new reservoirs of infection and may recontaminate domestic animals; examples include bovine tuberculosis in the United Kingdom associated with *Mycobacterium bovis* infection in badgers (*Meles meles*) (*18*) and brucellosis in outdoor-reared swine in Europe that resulted from spillover from the wild boar brucellosis (*Brucella suis* biovar 2) reservoir (*19*).

## Wildlife Trade and Translocation

Wildlife trade provides mechanisms for disease transmission at levels that not only cause human disease outbreaks but also threaten livestock, international trade, rural livelihoods, native wildlife populations, and ecosystem health (*7*). Worldwide, an estimated 40,000 primates, 4 million birds, 640,000 reptiles, and 350 million tropical fish are traded live each year (*7*). International wildlife trade is estimated to be a US $6-billion industry (*20*).

Translocation of wild animals is associated with the spread of several zoonoses. Rabies was introduced in the mid-Atlantic states in the 1970s when hunting pens were repopulated with raccoons trapped in rabies-endemic zones of the southern United States (*21*). In Eastern Europe, raccoon dogs (*Nyctereutes procyonoides*) are becoming a new reservoir for rabies, in addition to the established red fox reservoir, as raccoon dogs have spread into new habitats from accidental release of animals raised for fur trade (*22*). Brush-tailed possums (*Trichosurus vulpecula*) from Tasmania were introduced into New Zealand to establish a new species of fur-bearing animals. The translocated population proliferated and is now estimated to be >70 million, of which 3%–30% are possibly infected by *M. bovis*, a permanent threat to the cattle- and deer-farming industries (*21*). Translocation of hares from central and Eastern Europe for sporting purposes led to several outbreaks of tularemia, introduction of *B. suis* biovar 2 to western Europe, and subsequent encroachment of this brucellosis strain into the wild boar population of western Europe (*19*). During 1993–2003, *B. suis* biovar 2 infections were reported in >40 outdoor-rearing pig farms in France (*19*).

Illegal trade can also be a possible source of human infection. In March 1994, psittacosis developed in several customs officers in Antwerp, Belgium (*23*). A customs officer had been hospitalized with pneumonia 10 days after exposure to parakeets illegally imported by an Indian sailor. The risk of contracting psittacosis was 2.8× higher for officers exposed to parakeets >2 hours than for those exposed only briefly. Similarly, a highly pathogenic avian influenza A H5N1 virus from crested hawk eagles smuggled into Europe by air travel has been isolated and characterized (*24*); fortunately, however, screening of human and avian contacts indicated that no dissemination had occurred.

## Bushmeat, Wet Markets, Exotic Foods, and Zoonotic Diseases

Another risk factor related to the emergence of zoonotic diseases from wildlife has been the considerable increase in consumption of bushmeat in many parts of the world, especially Central Africa and the Amazon Basin, where 1–3.4 million tons and 67–164 million kilograms, respectively, are consumed each year (*7*). The simian foamy virus has been identified as a zoonotic retrovirus that infects people who have direct contact with fresh nonhuman primate bushmeat; this finding indicates that such zoonoses are more frequent, widespread, and contemporary than previously appreciated. Similarly, new retroviruses, human T-lymphotropic virus types 3 and 4 were found in persons who hunt, butcher, or keep monkeys or apes as pets in southern Cameroon (*25*). The combination of urban demand for bushmeat (a multibillion-dollar business) and greater access to primate habitats provided by logging roads has increased the amount of hunting in Africa, which has increased the frequency of human exposure to primate retroviruses and other disease-causing agents. Similarly, several outbreaks of Ebola virus in western Africa have been associated with consumption of bushmeat, mainly chimpanzees that were found dead (*26*).

Traditional and local food markets in many parts of the world can be associated with emergence of new zoonotic diseases. Live animal markets, also known as wet markets, have always been the principal mode of commercialization of poultry and many other animal species. Such markets, quite uncommon in the United States and, until recently, in California, are emerging as a new mode of commercialization within specific ethnic groups for whom this type of trade assures freshness of the product but raises major public health concerns. The avian influenza epidemic, which began in Southeast Asia in 2003 and recently spread to other parts of the world, is directly related to infected birds sold live in traditional markets. Live bird markets facilitate the spread of this avian H5N1 virus by wild birds (*27*). Similarly, the newly discovered severe acute respiratory syndrome–associated coronavirus was linked to trade of live, wild carnivores, especially civets, in the People’s Republic of China (*2*). However, recent data suggest that civets may be only amplifiers of a natural cycle involving trade and consumption of bats (*28*). Trichinellosis has long been associated with consumption of undercooked meat from wild animals, such as bears, and now consumption of uncooked meat from deer and wild boar has recently been associated with emergence of severe cases of hepatitis E in hunters in Japan (*29*). Industrialized nations’ new taste for exotic food has also been linked with various zoonotic pathogens or parasites, such as protozoa (*Toxoplasma*), trematodes (*Fasciola* sp., *Paragonimus* spp.), cestodes (*Taenia* spp., *Diphyllobothrium* sp.), and nematodes (*Trichinella* spp., *Anisakis* sp., *Parastrongylus* spp.).

## Ecotourism

Adventure travel is the largest growing segment of the leisure travel industry; growth rate has been 10% per year since 1985 (Adventure Travel Society, pers. comm.). This type of travel increases the risk that tourists participating in activities such as safaris, tours, adventure sports, and extreme travel will contact pathogens uncommon in industrialized countries. The most commonly encountered rickettsial infection in travel medicine is African tick bite fever, caused by *Rickettsia africae* and transmitted in rural sub-Saharan Africa by ungulate ticks of the *Amblyomma* genus; >350 imported cases have been reported from several continents during the past few years (*30*). Most patients are infected during wild game safaris and bush walks. Moreover, because ecotourism is becoming increasingly popular with international travelers, more cases of imported rickettsioses are likely to occur in Europe, North America, and elsewhere in years to come.

*Cercopithecine herpesvirus* 1 (herpes B virus) is an alpha herpesvirus endemic to Asian macaques, which mostly carry this virus without overt signs of disease. However, zoonotic infection with herpes B virus in humans usually results in fatal encephalomyelitis or severe neurologic impairment (*31*). Herpes B virus has been implicated as the cause of ≈40 cases of meningoencephalitis in persons who had direct or indirect contact with laboratory macaques. A survey of workers at a Balinese Hindu temple, a major tourist attraction where macaques roam free, showed that contact sufficient to transmit B virus occurred commonly between humans and macaques. Furthermore, 31 (81.6%) of 38 macaques at that location had antibodies to herpes B virus (*31*).

## Petting Zoos and Exotic Pets

Petting zoos, where children are allowed to approach and feed captive wildlife and domestic animals, have been linked to several zoonotic outbreaks, including infections caused by *Escherichia coli* O157:H7, salmonellae, and *Coxiella burnetii* (*32*). More than 25 outbreaks of human infectious diseases associated with visitors to animal exhibits were identified during 1990–2000 (*32*). In an outbreak of salmonellosis at a Colorado zoo, 65 cases (most of them in children) were associated with touching a wooden barrier around the Komodo dragon exhibit. *Salmonella* organisms were isolated from 39 case-patients, a Komodo dragon, and the wooden barrier. Children who did not become infected were more likely to have washed their hands after visiting the exhibit (*33*).

Exposure to captive wild animals at circuses or zoos can also be a source of zoonotic infection. Twelve circus elephant handlers at an exotic animal farm in Illinois were infected with *M. tuberculosis*, and 1 had signs consistent with active disease after 3 elephants died of tuberculosis. Medical history and testing of the handlers indicated that the elephants had been a probable source of exposure for most of the infected persons (*34*). After an *M. bovis* outbreak in rhinoceroses and monkeys at a zoo in Louisiana, 7 animal handlers, previously negative for tuberculosis, had positive test results (*35*).

Exotic pets are also a source of several human infections that vary from severe monkeypox related to pet prairie dogs or lyssaviruses in pet bats to less severe but more common ringworm infections acquired from African pygmy hedgehogs or chinchillas. Epidemiologic and animal trace-back investigations confirmed that the first community-acquired cases of monkeypox in humans in the United States (71 cases) resulted from contact with infected prairie dogs that had been housed or transported with African rodents imported from Ghana (*3*). Similarly, an outbreak caused by *Francisella tularensis* type B occurred among wild-caught, commercially traded prairie dogs; *F. tularensis* antibodies in 1 exposed person documented the first evidence of tularemia transmission from prairie dog to human (*36*). African pygmy hedgehogs have been implicated in human salmonellosis cases in the United States and Canada (*37*). In the United States, the number of commercialized reptiles, especially iguanas, imported per year has increased considerably to ≈1 million. The number of human cases of salmonellosis, especially in very young children, increased dramatically in parallel with iguana pet ownership. The Centers for Disease Control and Prevention estimates that ≈7% of human infections with salmonellae in the United States are associated with having handled a reptile. Most iguanas have a stable mixture of *Salmonella* serotypes in their intestinal tract and intermittently or continuously shed *Salmonella* organisms in their feces (*38*).

Eight cases of rabies caused by a new rabies virus variant were reported in the state of Ceará, Brazil, from 1991 through 1998. Marmosets (*Callithrix jacchus jacchus*) were determined to be the source of exposure. These primates are common pets; most cases occurred in persons who had tried to capture them, and 1 case was transmitted by a pet marmoset (*39*). In 1999, encephalitis was diagnosed in an Egyptian rousette bat (*Rousettus egyptiacus*) that had been imported from Belgium and sold in a pet shop in southwestern France. The pet bat was infected with a Lagos bat lyssavirus and resulted in the treatment of 120 exposed persons (Y. Rotivel, pers. comm.).

## Conclusion

Emerging infectious diseases have a major effect on human health and can create tremendous economic losses. Animals, particularly wild animals, are thought to be the source of >70% of all emerging infections (*40*). Leading factors for emergence of zoonoses are unbalanced and selective forest exploitation and aggressive agricultural development associated with an exponential increase in the bushmeat trade (*8*). Similarly, the increase of ecotourism, often in primitive settings with limited hygiene, can be associated with the acquisition of zoonotic agents. Therefore, development of appropriate programs for surveillance and for monitoring emerging diseases in their wildlife reservoirs is essential. Most animal pathogens for which surveillance programs exist relate to farm animals, and few or no programs are specifically aimed at wildlife. Two different but complementary approaches are 1) to monitor the presence of specifically identified pathogens that have emerged as human pathogens and 2) to investigate in a given wildlife species the presence of known or unknown infectious agents. Furthermore, conservation of habitat biodiversity is critical for preventing emergence of new reservoirs or amplifier species. Key measures for reducing the dispersion of emerging zoonoses include sustainable agricultural development, proper education of tourists about the risks of outdoor activities, and better control of the live animal trade (exotic pets, wet markets, bushmeat). Public health services and clinical practitioners (physicians, veterinarians) need to more actively educate the public about the risks of owning exotic pets and adopting wild animals.

As suggested by Kuiken et al. (*40*), it is time to form “a joint expert working group to design and implement a global animal surveillance system for zoonotic pathogens that gives early warning of pathogen emergence, is closely integrated to public health surveillance and provides opportunities to control such pathogens before they can affect human health, food supply, economics or biodiversity.” Major tasks that should be taken by the international community include better integration and coordination of national surveillance systems in industrialized and developing countries; improved reporting systems and international sharing of information; active surveillance at the interface of rural populations and wildlife habitats, especially where poverty and low income increase risks for pathogen transmission; training of professionals, such as veterinarians and biologists, in wildlife health management; and establishment of collaborative multidisciplinary teams ready to intervene when outbreaks occur.
